# Susceptibility and tolerance of rice crop to salt threat: Physiological and metabolic inspections

**DOI:** 10.1371/journal.pone.0192732

**Published:** 2018-02-28

**Authors:** Nyuk Ling Ma, Wan Afifudeen Che Lah, Nisrin Abd. Kadir, Mohamad Mustaqim, Zaidah Rahmat, Aziz Ahmad, Su Datt Lam, Mohd Razi Ismail

**Affiliations:** 1 School of Fundamental Science, Universiti Malaysia Terengganu, Kuala Terengganu, Terengganu, Malaysia; 2 Department of Biotechnology and Medical Engineering, University Technology Malaysia, Skudai, Johor, Malaysia; 3 School of Biosciences and Biotechnology, Faculty of Science and Technology, University Kebangsaan Malaysia, Bangi, Selangor, Malaysia; 4 Institute of Structural and Molecular Biology, Division of Biosciences, University College London, Gower Street, London, United Kingdom; 5 Institute of Tropical Agriculture, Universiti Putra Malaysia, Serdang, Selangor, Malaysia; Huazhong Agriculture University, CHINA

## Abstract

Salinity threat is estimated to reduce global rice production by 50%. Comprehensive analysis of the physiological and metabolite changes in rice plants from salinity stress (i.e. tolerant versus susceptible plants) is important to combat higher salinity conditions. In this study, we screened a total of 92 genotypes and selected the most salinity tolerant line (SS1-14) and most susceptible line (SS2-18) to conduct comparative physiological and metabolome inspections. We demonstrated that the tolerant line managed to maintain their water and chlorophyll content with lower incidence of sodium ion accumulation. We also examined the antioxidant activities of these lines: production of ascorbate peroxidase (APX) and catalase (CAT) were significantly higher in the sensitive line while superoxide dismutase (SOD) was higher in the tolerant line. Partial least squares discriminant analysis (PLS-DA) score plots show significantly different response for both lines after the exposure to salinity stress. In the tolerant line, there was an upregulation of non-polar metabolites and production of sucrose, GABA and acetic acid, suggesting an important role in salinity adaptation. In contrast, glutamine and putrescine were noticeably high in the susceptible rice. Coordination of different strategies in tolerant and susceptible lines show that they responded differently after exposure to salt stress. These findings can assist crop development in terms of developing tolerance mechanisms for rice crops.

## Introduction

Sodium (Na^+^) is an abundance element in the earth. Ocean itself make up of 71% of the surface of earth and hence it is not surprising that most plants will get in contact with Na^+^ at their growth phase. Agricultural practices (i.e. fertilizer application, poor irrigation systems coupled with increasing sea levels) have led to poor water quality and saline soil conditions [1]. Rice, a glycophyte, is very susceptible to saline soil. Prolonged exposure to saline conditions causes stress in these plants and leads to a significant decrease in grain production [2].

Plants that are in stress usually suffer from an imbalance in cellular homeostasis, due to the production of high-energy state successive electrons. The conversion of these successive electrons, transfers free electrons to O_2_ molecules which leads to the formation of reactive oxidative species (ROSs) [3]. ROSs, that normally act as signalling molecule, may induce oxidative damage at high concentration (i.e. chlorophyll loss, membrane lipid peroxidation, protein carbonylation, inactivating sulfhydryl (–SH) containing enzymes, nucleic acid (DNA) and cell membrane properties) and reduce the rate of photosynthesis by 50% [4].

Therefore, plants utilise an effective ROS scavenging mechanism through the catalytic action of antioxidants to maintain their homeostasis [2,5]. Nevertheless, some members of ROS were also found to act as signalling molecules for the activation of defence mechanisms in tolerant plants under stress conditions. Increase functioning of signalling molecules in tolerant line contributes to maintain homeostasis that resists environmental stress [6,7].

In recent years, several transcription factors were tested in order to improve salinity tolerance in rice such as AtHKT1;1 [8] and OsMYB48-1 [9]. However, the inspection of physiological aspects in relation to metabolome of the same variety of rice plants under saline conditions were relatively limited. One of the ultimate goals in plant system biology is to reveal the genotype-phenotype relationship in plant cellular systems, e.g. the metabolite deviation levels that correlate with biomass production. Therefore, by using plants with the same genetic background, the data obtained is important for trait prediction and therefore provides the opportunity to improve the breeding program. In this study we focused on rice hybrid lines with contrasting behaviours in response to salt stress. We examined differences in antioxidant activities, chlorophyll and water status, that cause metabolomic flux within plant cells. Nuclear magnetic resonance (NMR) enables rapid profiling of metabolomics flux during salt stress. We explored the correlations of metadata with physiological and biochemical changes.

## Materials and methods

### Screening of salinity susceptible and tolerant line

Seeds of rice (*Oryza sativa* L.) were obtained from the International Rice Research Institute (IRRI). A total of 46 genotypes entry of IRRI 31^st^ IRSSTN-SS1 and 46 genotypes entry of 31^st^ IRSSTN-SS2 were tested for their response to saline conditions by treating their seeds with 100 mM NaCl for 76 hours during the seed germination stage. Only the quality seeds that germinated well were then grown in normal soil conditions and their growth performance (i.e. number of lateral roots, length of the roots and shoot production) was recorded after 2 months.

### Micropropagation and salinity treatment

Seeds from the most tolerant and the most susceptible lines were selected for further analysis. Seeds were dehusked and washed thoroughly with tap water. The seeds were sterilized with 70% (v/v) ethanol for 2–3 minutes and rinsed with sterile distilled water. The seeds were then subsequently, sterilized with 50% (v/v) Clorox for 45 minutes. The seeds were then grown in culture condition (i.e. 70–85% relative humidity; 24 ± 2°C; photoperiod 16 h—8 h photosynthetic photon flux density of 250–350 μmol m^-2^ s^-1^) [10]. Germinated seeds were propagated by cutting hypocotyls and grown on MS medium supplemented with 2 mg/L 6-Benzylaminopurine. Explants were grown for 3 weeks in the culture room or until they reached approximately 5 cm. The seedlings were transplanted into rooting media supplemented with 8% sucrose and grown for another 3 weeks. Explants that were 12 cm in height were treated with different concentrations of NaCl (0 mM, 50 mM and 150 mM). Leaf and root tissue were harvested at different time frames (0 h, 8 h, 16 h, 32 h and 64 h) for analysis purposes.

### Determination of antioxidative enzyme activities

Enzymatic antioxidative defence mechanisms under salinity stress were assessed by examining major antioxidants such as catalase (CAT), ascorbate peroxidase (APX) and superoxide dismutase (SOD). For the CAT enzyme activity analysis, samples were ground in 50 mM phosphate buffer (pH 7.4) and centrifuged at 10 000 g, 4°C for 10 minutes. The supernatant was then mixed with reaction buffer (19 mM H_2_O_2_ in 50 mM phosphate buffer, pH 7.0) and the reaction was spectrophotometrically read at 240 nm comparing the composition to H_2_O_2_ (ε = 39.4 mM−1 cm−1) [11].

To detect the APX activity, leaf samples were ground in buffer (0.1 M phosphate buffer, pH 7.0; 1 mM ascorbic acid) and centrifuged at 10 000 g, 4°C for 10 minutes. The supernatant was then mixed with 3 ml of reaction buffer (100 mM phosphate buffer (pH 7.0); 3 mM ascorbic acid; 3 mM EDTA and 1.5 mM H_2_O_2_) and its activities were spectrophotometrically read at 290 nm (ε = 2.8 mM−1 cm−1) [12].

To determine the activity of SOD, leaves were ground in buffer (50 mM sodium phosphate buffer pH 7.5, 0.5 mM EDTA.Na_2_) and centrifuged at 14,000 g for 30 minutes at 4°C. The supernatant was then mixed with reaction buffer (10 μL of 6 mM NBT, 100 μL of 40 mM xanthine, 890 μL of 50 mM KH_2_PO_4_/K_2_HPO_4_, pH 7.5). The reaction was started by adding 0.025 U of xanthine oxidase followed by the reduction of NBT and the activity is measured at 560 nm [13].

### Observation of physiological changes

Ion content in the root was measured by mixing 0.05 g of root sample with 20 ml of distilled water [14]. Samples were incubated in water bath at 95°C for 1 hour and subsequently autoclaved at 121°C for 20 minutes. The sodium and potassium content were determined using the atomic absorption spectrophotometer [15]. The chlorophyll content in the leaf was extracted using methanol and the absorbance was measured using a spectrophotometer at 665.2 nm and 652 nm [16]. To calculate the relative water content (RWC) in leaves during salinity stress, leaves were cut into 2 cm long and its fresh weight was measured immediately. The leaves were then placed in distilled water for 24 hours in dark conditions. Next day, the leaves were blotted dry with filter paper and their turgid weight was measured. The dry weight was then recorded after constant weight was obtained after being dried at 50°C. RWC were calculated using formula below:

$$RWC\;(\%)=\frac{fresh\;weight-dry\;weight}{turgid\;weight-dry\;weight}\times 100$$

### Metabolites extraction for NMR analysis

Plant samples were ground with liquid nitrogen by using a prechilled mortar and pestle then freeze dried for 24 h at -20°C and pressure set at 1.0324 mbar). The samples were then mixed with 1 ml aqueous solvent, prepared by adding methanol and water with ratio 1:1. Samples were then vortexed for 5 minutes before adding 1 ml of 100% chloroform and vortexed again for 5 minutes. Mixtures were centrifuged at 10,000 g at 4°C for 15 minutes. Aqueous and chloroform layers were collected separately into glass vials and air-dried in a fume hood for 24 h. Aqueous samples were stored at -80°C and water was completely dried up by freeze dried at -20°C and pressure set at 1.0324 mbar).

### NMR data acquisition

Plant extract by aqueous solvent was resuspended in 600 μl of phosphate buffer (0.1 M, pH 7) containing 10% of Deuterium oxide (D_2_O), while chloroform extracted sample was resuspended in 600 μL of absolute chloroform-d4 containing 0.01% of tetramethylsilane (TMS) (Sigma Aldrich). The mixtures were transferred into 5 mm NMR tube for data acquisition using NMR (Bruker DRX-400, Fremont, CA). The temperature of program was maintained at 300 K. 1D NMR (64 K FID data points) spectra was acquired through parameter 9-1s (60) pulse, 6-kHz spectral width, 5-s relaxation delay with presaturation residual water resonance and 100 transients were collected into 64 k data points and exponential line-broadenings of 0.8 Hz were applied before the Fourier transformation. Correlation spectroscopy-double quantum filtered (COSY-DQF), heteronuclear multiple quantum correlation (HMQC) and hetero multiple bond correlation (HMBC) experiments were used for spin system assignment.

### NMR data processing and statistical analysis

^1^H NMR spectra were acquired and aligned using the MestReNova 9.0 software (Mestrelab Research, Escondido, CA, USA). NMR spectra were normalized, phased and baseline corrected (Polynomial Fit method) before imported into SIMCA-12 (Umetrics, Umea, Sweden). Chloroform extract were referenced to TMS peaks (0.00 ppm), aqueous were referred to alanine (1.47 ppm). UV scaling was applied to the data and a score plot was generated to describe the data using PLS-DA. NMR peaks were then manually identified according to integration, chemical shift and peaks multiplicity by comparing to online databases such as Human Metabolome Database (HMDB) [17], Biological Magnetic Resonance (BMRB) [18] and Yeast Metabolome Database (YMDB) [19]. Fold changes between metabolites in treatment (tolerant vs control; susceptible vs control) at different time points respectively were detected by comparing average of spectra intensity from three biological replicates with t-test P<0.05. Statistical significance for antioxidant, RWC and ions were determined by ANOVA with Tukey HSD multiple comparison post-test with significant value of P<0.05.

## Results and discussion

### Screening of salt susceptible and tolerant line

**Fig 1:**
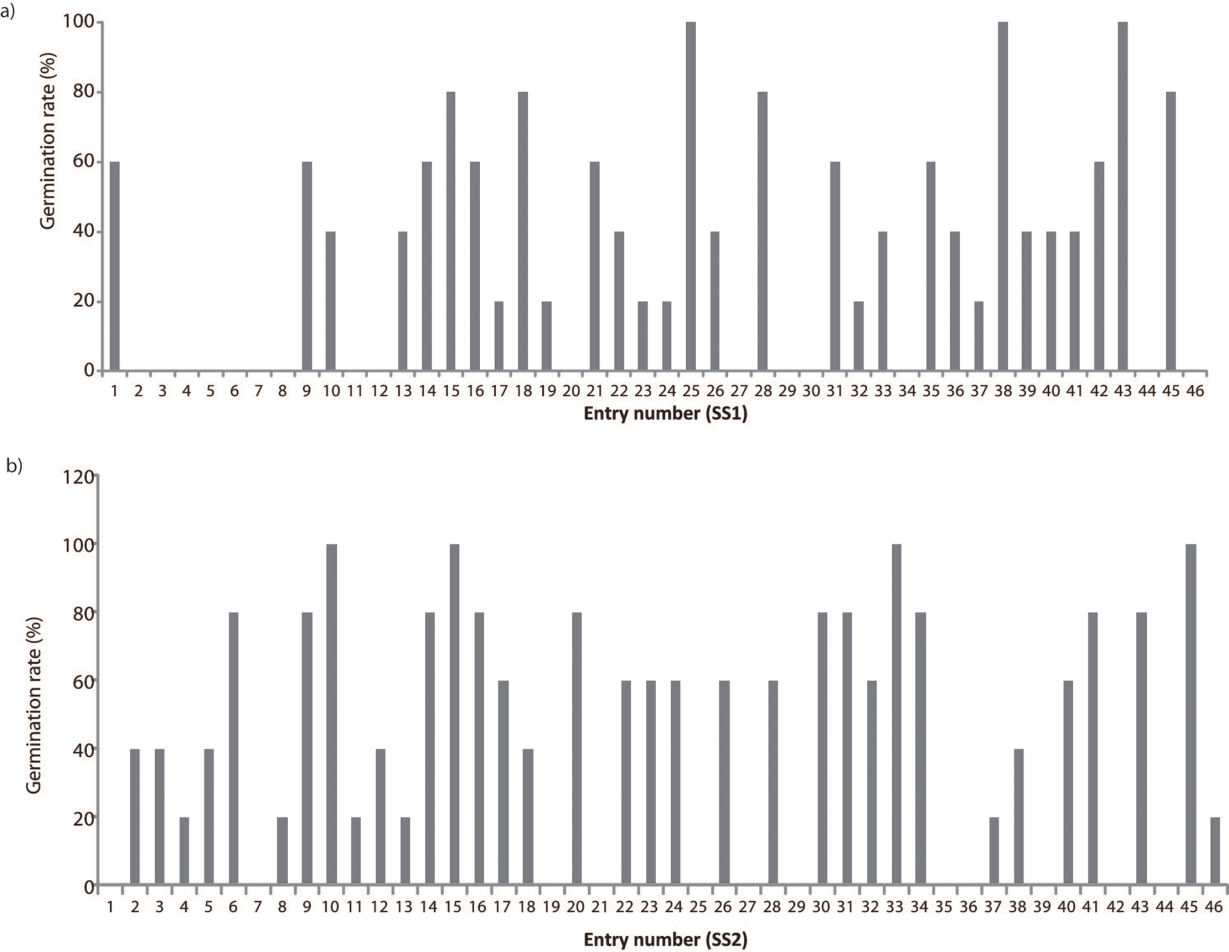
Percentage of germination from the total of 46 genotypes of a) IRSSTN-SS1 line and b) IRSSTN-SS2 line under 100 mM NaCl treatment.

**Fig 2:**
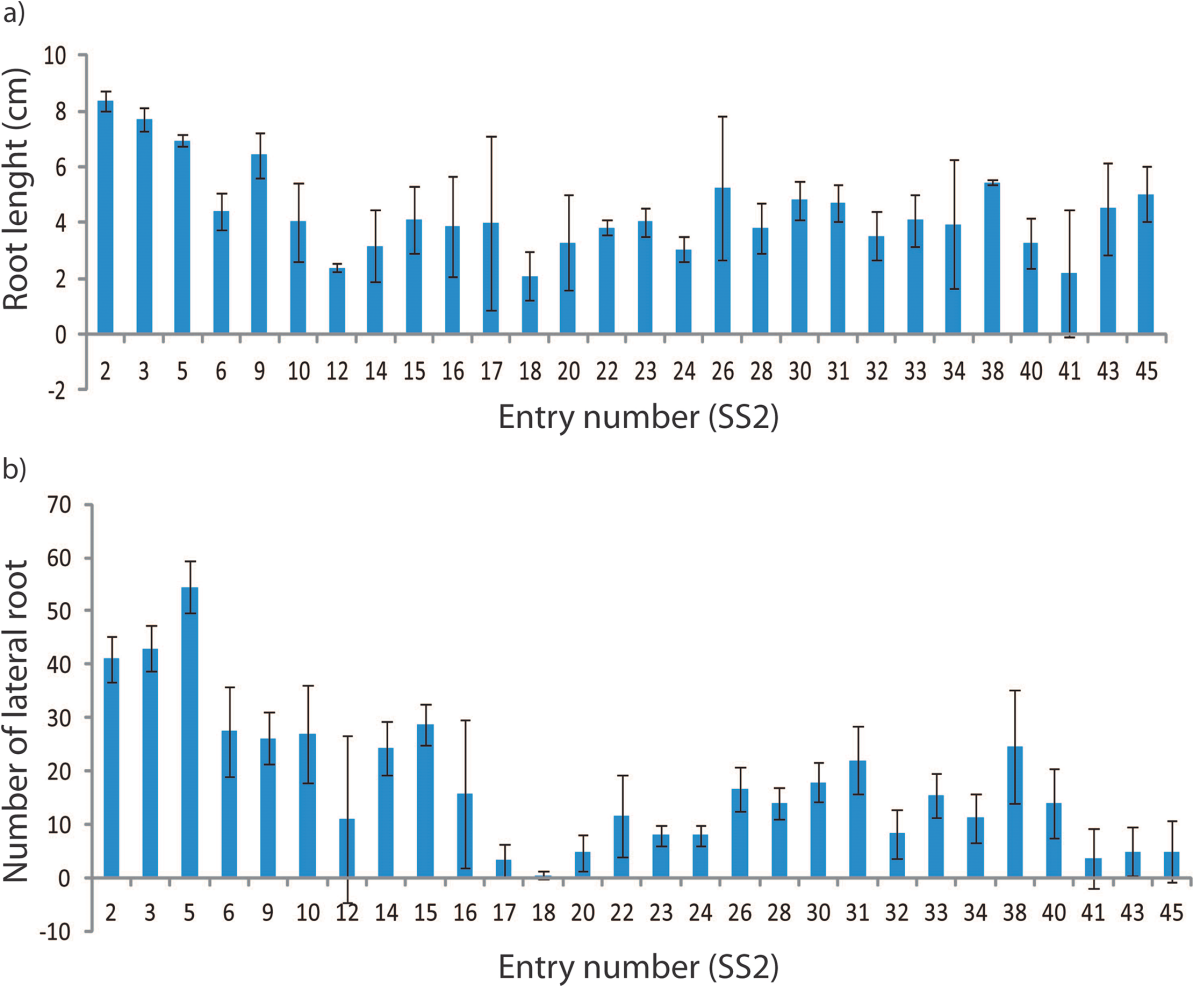
a) Root length and b) number of lateral root produced from IRSSTN-SS1 line and IRSSTN-SS2 line following 72 h of salinity (50 mM NaCl) treatment.

Prior to salinity treatment, rice lines were selected based on the germination rate of five biological individuals after salinity treatment. From the total of 92 lines tested, only 64 were successfully germinated (Fig 1). The well-germinated seeds were subjected to salinity treatment and its length and number of lateral roots produced from each genotype were recorded (Fig 2). There were only 11 genotypes that have significantly longer and higher number of roots. These 11 genotypes were then compared to local drought tolerant variety (MR 219) [20] under salt treatment and its roots system were observed. Only 5 genotypes have longer root system compared to MR 219. We selected the most susceptible line (SS2-SS18) and most tolerant line (SS1-SS14) based on highest/lowest root length and number of lateral roots produced under salinity condition. The most susceptible line (SS2-SS18) and tolerant line (SS1-SS14) were selected for the following salt treatment.

### Antioxidant activities

**Fig 3:**
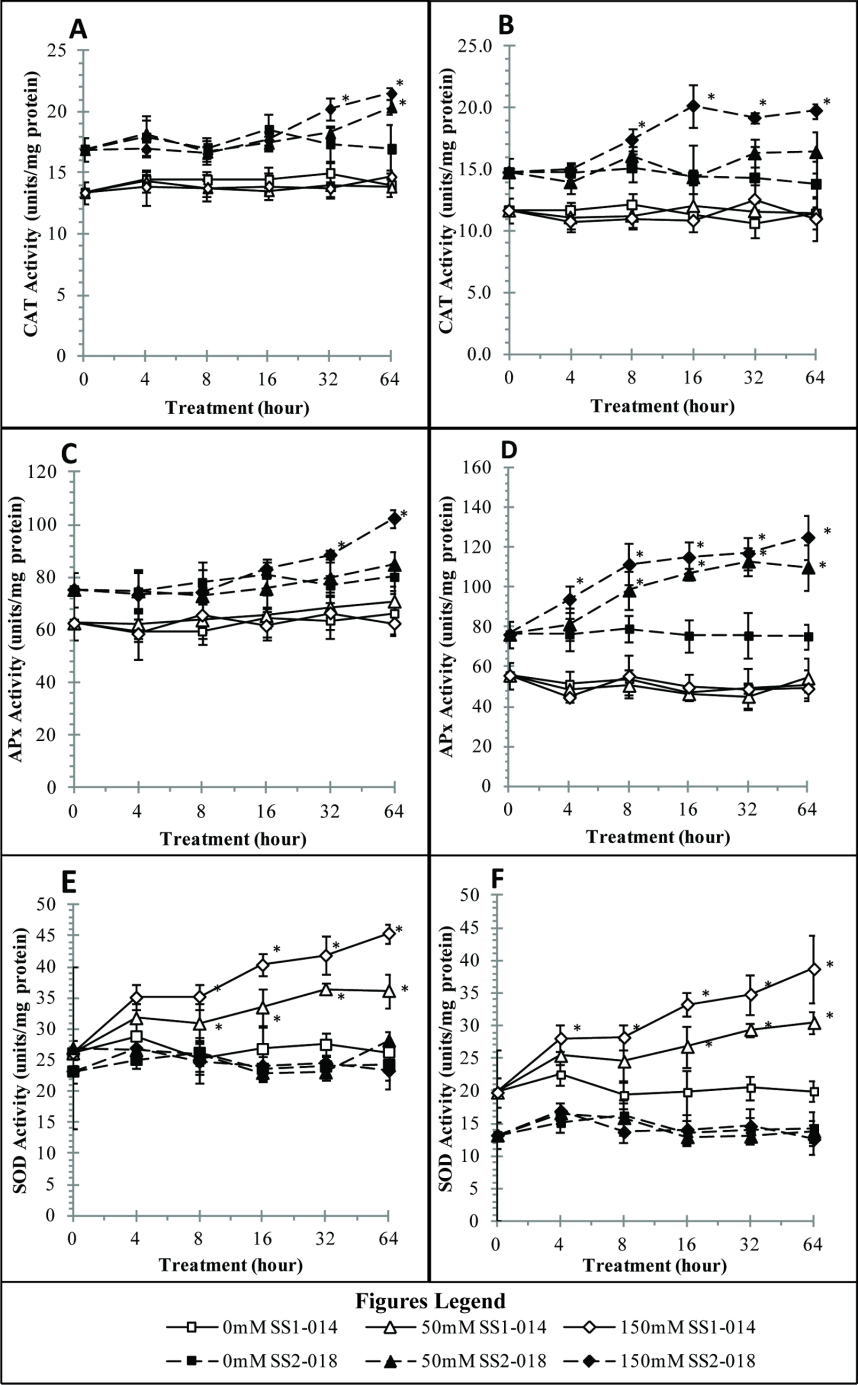
Antioxidant activities of tolerant (SS1-014) and susceptible (SS2-018) rice lines under control, low dose (50 mM NaCl) and high dose (150 mM NaCl) salinity stress. A) CAT leaf, B) CAT Root, C) APX leaf, D) APX root, E) SOD leaf and F) SOD Root. Asterisk (*) means significant changes between control and treated samples.

Examination of antioxidant activities provides some clue to the homeostasis condition and antioxidant defence against salinity stress [21–23]. When encounters with stress (e.g. salinity stress), ROSs will be produced and accumulated in plant cells. Plants induce oxidative stress tolerance by modulating the activities of antioxidant enzymes, to be more precise, these molecules are scavenged by SOD, CAT and APX [24]. Leaf samples in all salt treatment of susceptible line displayed higher CAT and APX activities compared to tolerant sample, with more distinctive activities observed in high dose of 64 h treatment (Fig 3A–3D). In contrary, the response of SOD was observed to be higher in salt tolerant plant and lower in salt susceptible plant (Fig 3E and 3F). Surprisingly, the whole salinity treatment did not trigger any CAT and APX activities in either leaf or root of tolerant line (Fig 3E and 3F). SOD has been designated as an important enzyme to combat ion imbalance. In addition, SOD is the first defence mechanism towards salinity stress as it has the ability to convert superoxide (O_2_^-^) radicals into more stable form, such as H_2_O_2_ [25], while CAT and APX are involved in the breakdown of H_2_O_2_ [26]. Unfortunately, accumulation of water (by-product of H_2_O_2_ breakdown) would dilute the concentration gradient within the plant body (i.e. causes the plant body to lose water content easier when more salt ions move into the plant) and possibly death by ion intoxication [2,21]. In this study, SOD was found to be drastically being produced in tolerance variety. Inability to increase SOD production as the primary response towards salinity exposure may contribute to higher oxidative stress due to inability to activate or amplify stress signalling pathways [5,27] and this may implies the breaking of balanced homeostasis following salt treatment. This result points towards a strong correlation between SOD production and inherent salt tolerant characteristics. SOD increment has also been reported in aluminium ion tolerant rice lines [28], cold tolerant rice lines [29] and salt-tolerant rice in prolonged saline stress [30].

### RWC, chlorophyll and Na^+^/K^+^ ratio

**Fig 4:**
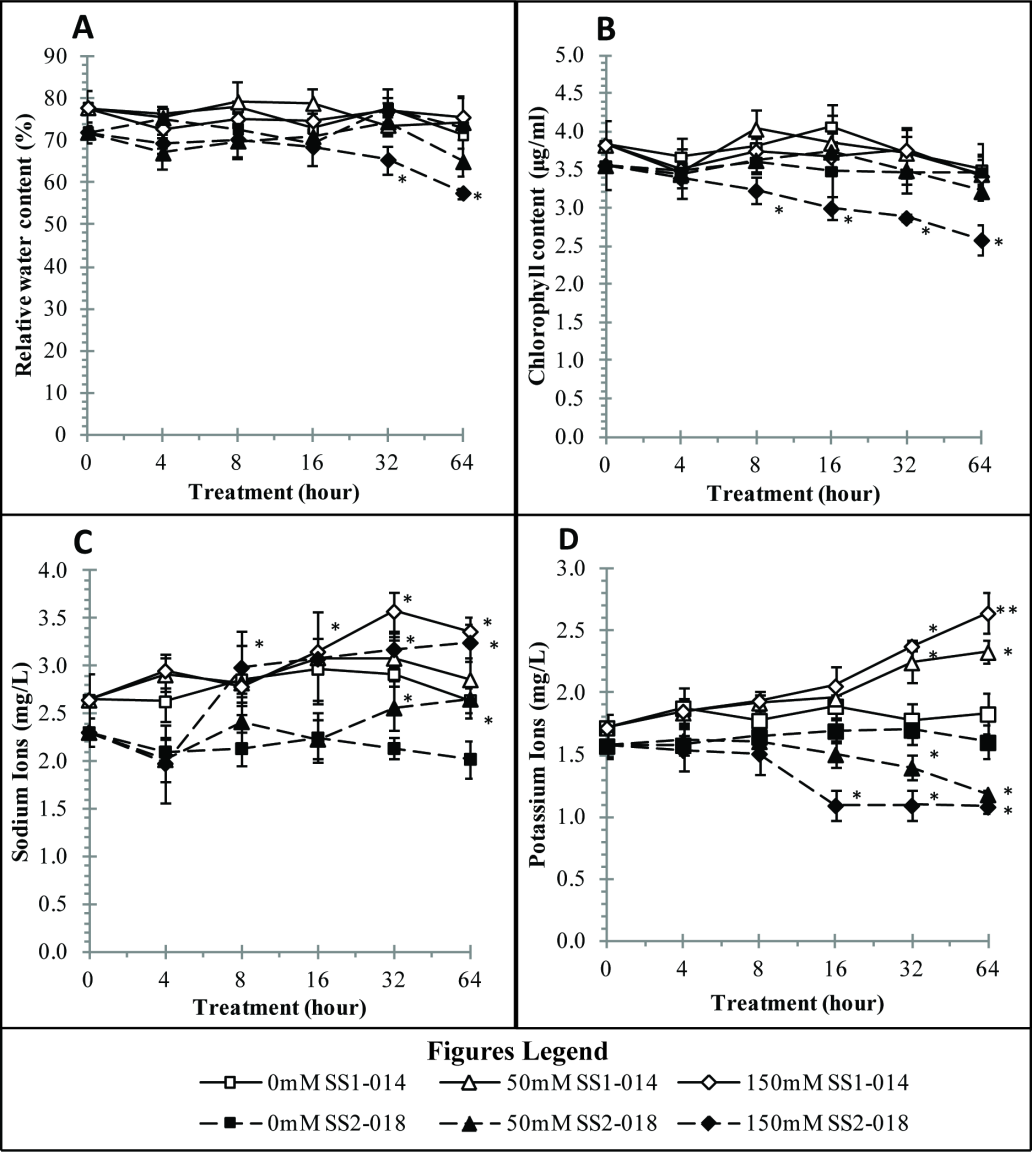
Physiological activities of tolerant (SS1-014) and susceptible (SS2-018) rice lines under control, low dose (50 mM NaCl) and high dose (150 mM NaCl) of salt stress condition. A) RWC in leaf, B) chlorophyll content in leaf, C) sodium ion content (Na^+^) in root (Na^+^), D) potassium ion content (K^+^) in root. Asterisk (*) means significant changes between control and treated samples.

RWC, chlorophyll content and ion content are important parameters to determine plant growth/physiological fitness in various growth conditions [23,31,32]. Healthy tolerant plants with low salt injury were found to maintain high RWC and chlorophyll content (Fig 4A and 4B). There were a reduction of 10% RWC and 25% chlorophyll content in susceptible plants compared to control after 8 h of salt treatment (Fig 4A and 4B). This suggest that the susceptible plants were in stress as early as 8 h of treatment. According to previous reports, RWC higher than 70% is needed for healthy plant development, while RWC lower than 60% is an indication of stress [33,34].

Low levels of Na^+^ ions have been shown to be beneficial to plants by improving the taste of many crops [35]. However, saline soils contain high amounts of Na^+^ ions which competitively inhibits the absorption of several similarly charged ions (K^+^, N^+^ and P^+^). These charged ions are important for physiological development and survival [36]. Na^+^ ions are transported into plant cells through osmotic stress when the water potential is lowered by inorganic Na^+^ and Cl^-^ ions. Low water potential creates logical responses in plant cells by direct ion uptake. Salt tolerance plants employ strategies such as reducing their net uptake of Na^+^, limiting Na^+^ translocation to the shoot and effective cellular partitioning. Nevertheless, salt susceptible species failed to do so results in translocation of large amounts of Na^+^ into shoot tissue leading to plant death [35]. In addition, competitive uptake of Na^+^ increases solute concentration within the root cells forces the water molecules to move out from root hair towards soil and creates water stress in plants. Salt stress levels in roots can be evaluated via ion content [32]. In general, it was observed that concentration of Na^+^ and K^+^ were higher in tolerant line, created a low Na^+^/K^+^ ratio. The Na^+^ and K^+^ increased simultaneously imply ion balance, i.e. the plants show little or no sign of stress. In contrast, lower K^+^ uptake when the Na^+^ concentration is high indicates the plants are under stress in susceptible plants (Fig 4C and 4D).

The ability of tolerate increased K^+^ under high Na^+^ concentration (in tolerant line) may due to the amount of expressed AKT1 K^+^ channels [37]. Rice plants with overexpression of protein AKT1 were observed to have better drought and osmotic stress tolerance through increased absorption of K^+^ in their tissues [37]. Tolerance mechanisms may have evolved to overexpress the number of AKT1 transporters under saline conditions in order to maintain higher amount of K^+^ in plant roots. Even though tolerant plants absorbed more Na^+^, they managed to maintain the content of other important ions such as K^+^ [38]. Tolerant plants have also been recorded to undergo time lapse adaption so that the plants reduce uptake of Na^+^ and increase K^+^ ions [39]. This behaviour of K^+^ ions under low and high saline treatment confirms the time lapse adaptation towards stress condition [40].

### Chemometric analysis and NMR profiling

**Fig 5:**
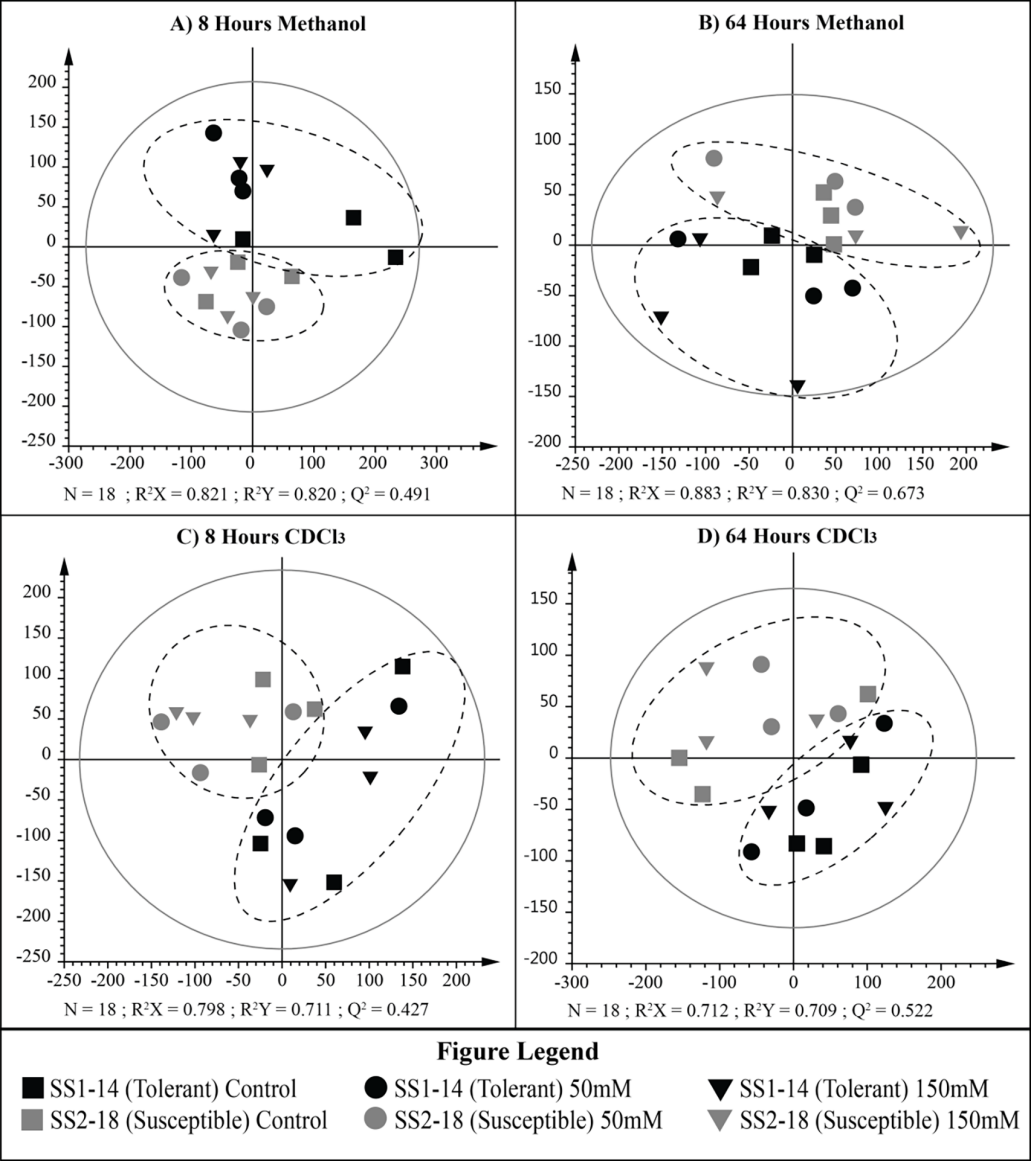
PLS-DA score plot of metabolic profile extracted from tolerant (SS1-014) and susceptible (SS2-018) rice lines following short-term NaCl treatment. (A) 8 h methanol extract, (B) 64 h methanol extract, (C) 8 h chloroform extract, (D) 64 h chloroform extract.

The PLS-DA result revealed a clear separation between tolerant and susceptible plants (Fig 5). PLS-DA was applied to discriminate between ^1^H NMR profiles arising from 1) between treatments and 2) among rice lines. In the four PLS-DA model, parameters of R^2^ Y and Q^2^ were, respectively, 0.711 and 0.427 (8 h chloroform), 0.709 and 0.522 (64 h chloroform), 0.8.20 and 0.491 (8 h aqueous) and 0.830 and 0.673 (64 h aqueous). These revealed the good discrimination and predictive ability of the models. We observed individuals from the same line were clustered together. These indicate that metabolite compositions are relatively homogenous within rice line and genotypic differences among rice lines are lower than variability in metabolites profile shown by incomplete discrimination in salt treatments.

### Potential salt stress biomarkers and pathway mapping

**Fig 6:**
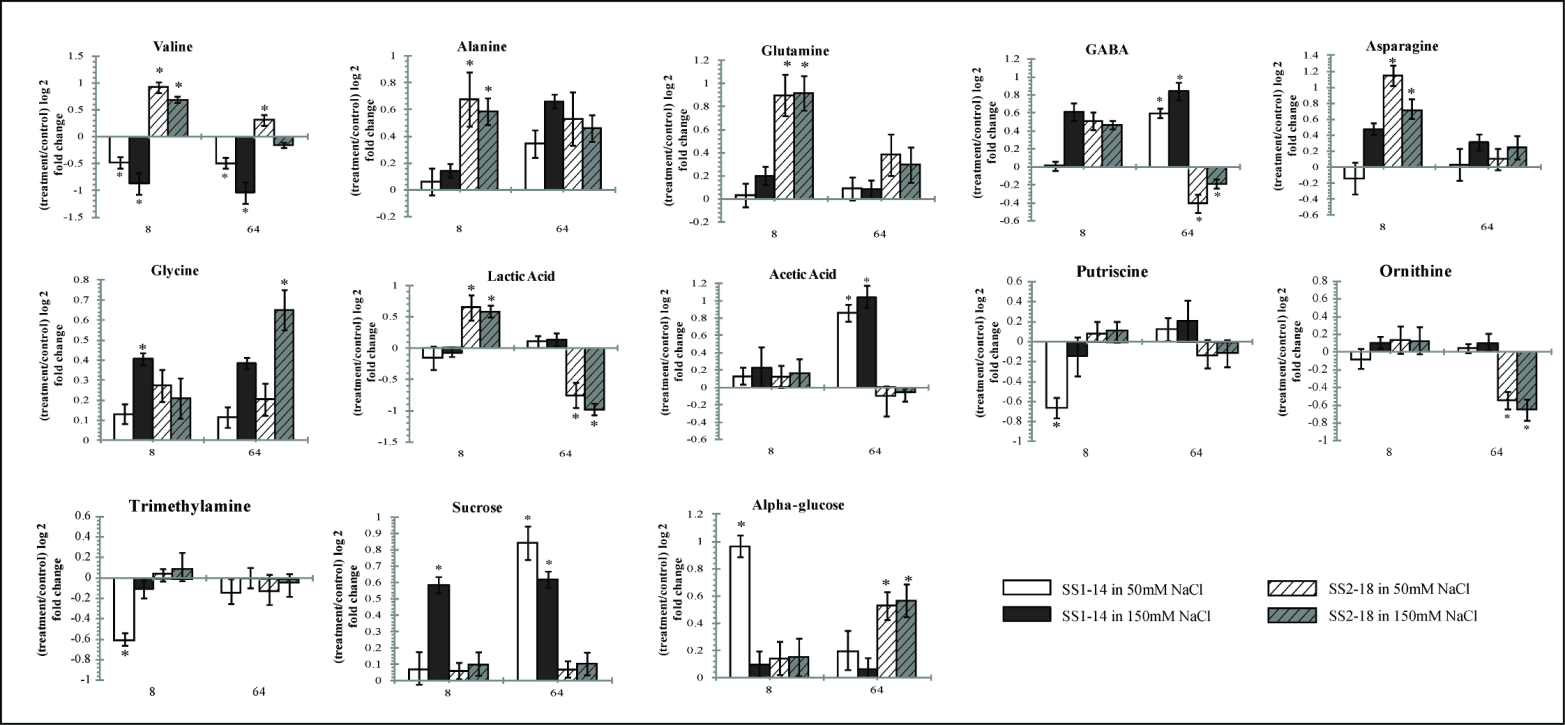
Fold changed bar graphs showing the relative levels of selected potential biomarkers for salinity stress obtained from aqueous extract. Asterisk (*) means significant changes between control and treated samples.

**Fig 7:**
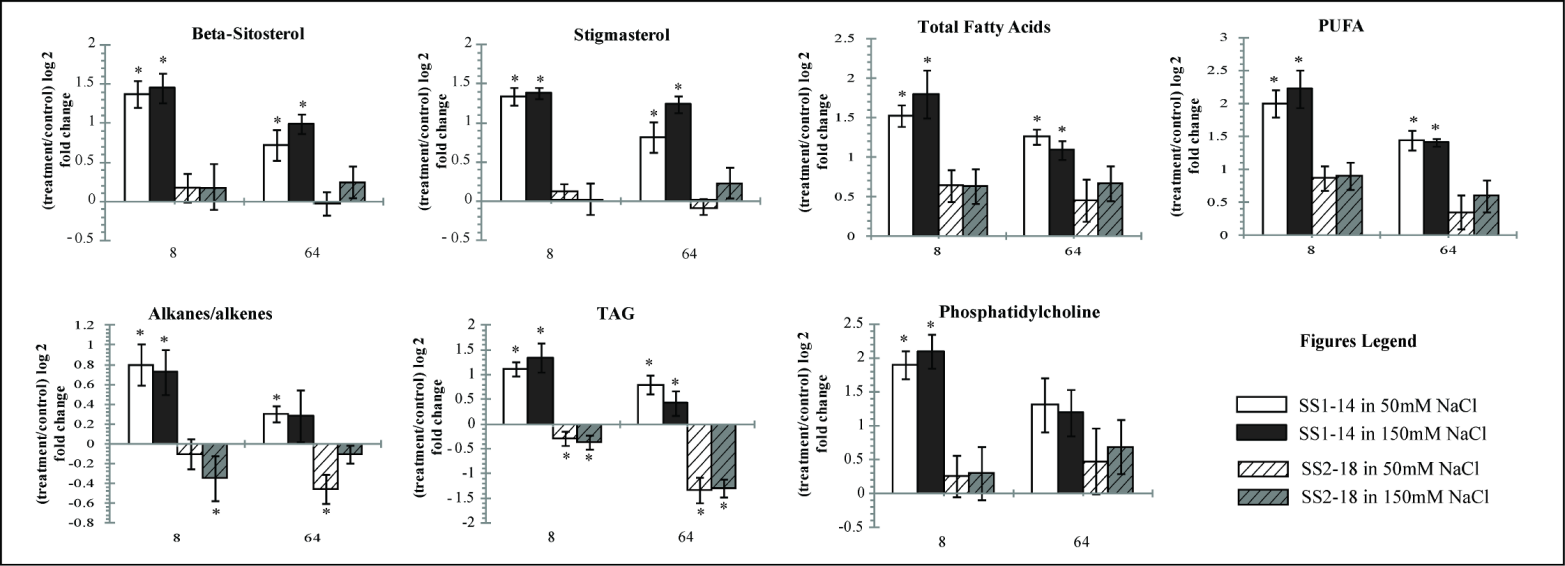
Fold changed bar graphs showing the relative levels of selected potential biomarkers for salinity stress obtained from chloroform extract. Asterisk (*) means significant changes between control and treated samples.

Identification of metabolites from ^1^H NMR spectra was done manually by matching ^1^H-NMR profiles (integration, chemical shifts and coupling constant) to known metabolites. The metabolites were confirmed by several 2D-NMR techniques and database tools such as HMDB, BMRB and BML(S1 Fig & S2 Fig). We identified a total of 26 metabolites (S1 Table & S2 Table) in this study and found 20 to be significantly different (T-test analysis, P<0.05). These metabolites, 13 identified from aqueous and 7 from chloroform, shown more than 50% change between tolerant and susceptible line (Fig 6 & Fig 7).

**Fig 8:**
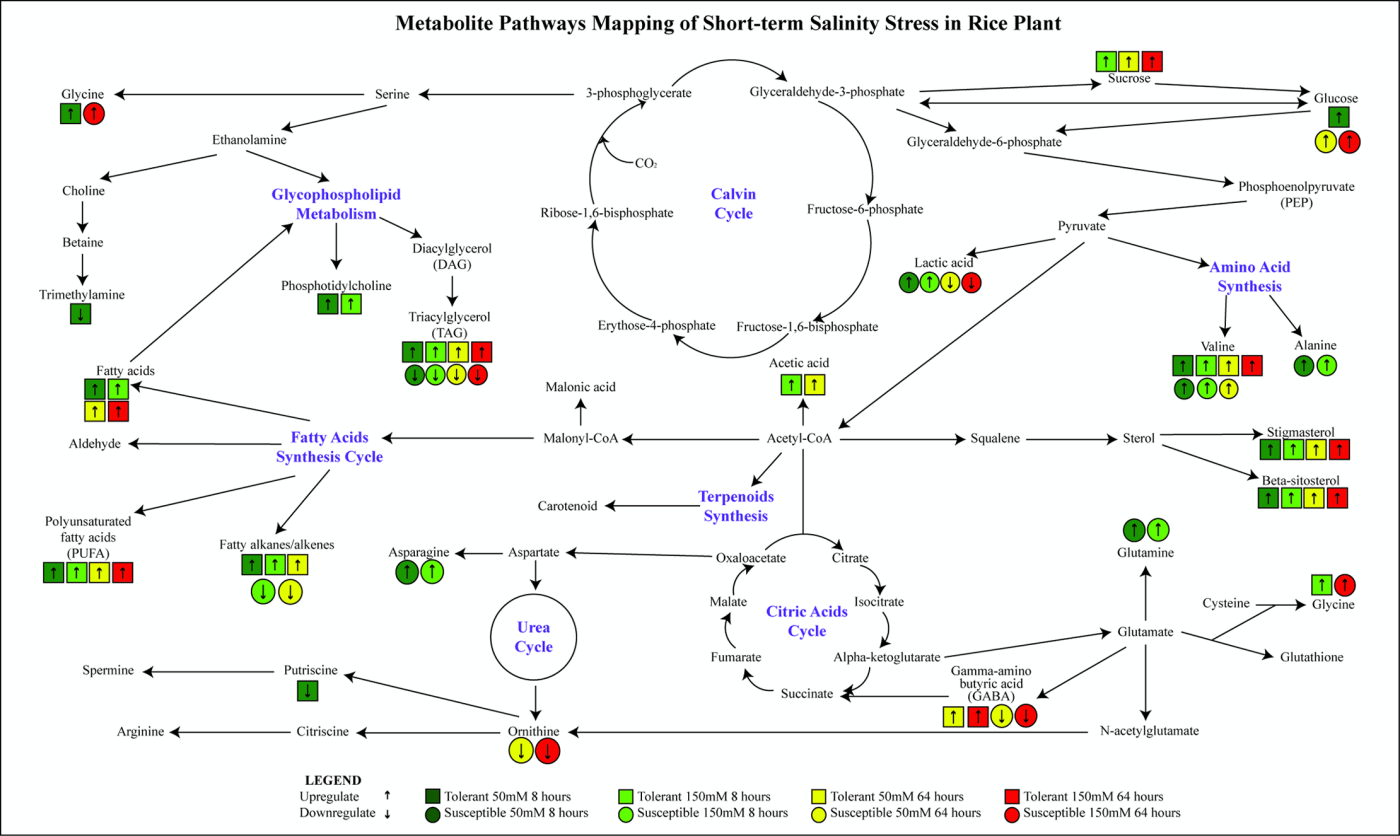
Changes of metabolites under short-term period (8 h and 64 h) exposure of low and high lose dosage of salt treatment in tolerant and susceptible rice plants.

All of the changed metabolites affected by salt stress were mapped to the four biological pathways below (Fig 8):

### Lipid biosynthesis pathway

Beta-sitosterol, stigmasterol and sterol were found to be significantly different between tolerant and susceptible lines. High accumulation of stigmasterols, beta-sitosterol, sterol, PUFA, alkyi chains, fatty acids, phosphotidylcholine, TAG and diglyceride were observed in tolerant rice line and were found higher at 8 h compared to 64 h treatment. Phosphatidylcholine has been reported to be elevated under short exposure of salt stress in rice, which attributed to membrane phosphorylation [41]. Another study using *Arabidopsis thalia L*. reported increment of choline head phospholipids concentration after exposure to salt and cold stress, suspected to be an important membrane-mediated cell signalling mechanism [42,43].

In addition, upregulation of fatty acids derivatives such as DAG, PUFA and free fatty acids remained a prominent response in tolerant rice which can be related to nonpolar and H_2_O_2_ wave interaction. Some reports suggest that lipid peroxidation may increase under stress as plants strategize by using PUFA to combat ROS [44,45]. In our study, accumulation of PUFA increased rapidly after short term exposure. This suggests that tolerant plants are in a preparatory phase to activate lipid peroxidation, to further detoxify ROS and reduce oxidative stress. Although the relationship between ROS signalling and activation of defence cascade reaction seems to be connected, further analysis on lipid peroxidation is needed to illustrate more concrete findings.

### Amino acids biosynthesis

Interestingly, amino acids such as alanine, glutamine and asparagine show immediate response and increased in susceptible plants as soon as 8 h in both low and high treatments (Fig 7). Accumulation of these metabolies has been reported in rice shoots and roots under salt stress acting as the nature stress regulators [2].

It is also worth mentioning that important metabolites such as GABA, acetic acid and choline that only increased in the tolerant line when treated with salt stress. Ramesh et al., 2015 reported that upregulation of GABA was an extension towards a more tolerant characteristic. They also speculated that acetic acids to be an intermediate metabolite, important for assistance in activation of stress cascade kinases reactions in higher plants [46]. GABA has been discussed to act as buffering mechanisms in carbon and nitrogen metabolism. High elevation of GABA levels can be deduced as intracellular signalling molecules in plants. However, the specific molecular responses are still unknown [33,47]. Therefore, it is suggested that not only a single metabolite, but an effective regulation and signalling of complex mechanisms was the main key reason to tolerant characters such as in barley and wheat [6,48,49]. However for ornithine, reports suggest that accumulation of ornithine can deduce more tolerant character for plants under abiotic stress [50,51].

### Polyamines and amines

Putrescine, methylamine and betaine were found reduced more than 0.5 fold in tolerant line 8 h after salt treatment. These results are in agreement with previous findings that putrescine levels decrease after treated with stress specifically in rice plant as an early response [52]. Production of polyamines such as putrescine is believed to assist in cell proliferation and differentiation in plant organs such as leaves. Polyamines have only recently been classified as a new group of phytohormone like plant regulators which important for maintaining cell growth and development [53]. The polyamines’ most notable effects have been observed at the basic of many cellular processes such as DNA replication, transcription, cell proliferation, modulation of enzymatic activities and maintaining ionic balance besides securing cell membranes and cell walls [54,53]. Elevated levels of polyamines have been detected in plants that have been exposed to drought (barley), salinity (mung bean, tomatoes, rice, and wheat), temperature (cucumber) and metal toxicity (rapeseed) stresses [55,54,53]. In a study conducted in rice, salt tolerant rice cultivars were able to increase levels of endogenous spermidine and spermine while its obligate precursor putrescine remained [54]. However both spermidine and spermine levels in salt susceptible cultivars were the exact opposite therefore suggesting an increased conversion rate of putrescine to important polyamines (spermidine and spermine) as a tolerant characteristic [54]. In addition, The positive effects of polyamines in conveying tolerance against stress, specifically salt stress have been affiliated with improved membrane integrity, increased regulation towards production of osmolytes and maintainance of Na+ and Cl- ion ratios [53].

Betaine and choline are closely related with each other in order to produce enough compatible solutes under salt stress. Previous reports have found that both compounds serve as biomarkers in tolerant plants under unfavourable conditions [56,57].

### Osmolyte

In our study, sucrose, mannitol and alpha-glucose were found increased in all treatments and all plant types. Sugars are known osmoprotectants which act as water reservoir protectors to maintain RWC in plants under stress conditions [3,58]. Sugar has generally been known to make the environment within the plant cell more concentrate [6,59]. However, the type of sugar produced varies from rice line to rice line due to different catabolism of carbohydrates and may point to differing cues for differentiating plants with tolerant characteristics. The question as to why plants with different tolerance towards stress choose to produce different sugar compounds is still poorly understood [60]. Compartmentalization of sugar was also different between rice plants. Tolerant plants majorly produced sucrose (disaccharides) as its major carbon store while susceptible plants majorly produced alpha-glucose (simplest class sugar). Sucrose is an important transported sugar in plant physiology. In a salty environment, several reports suggest that production of sugars by photosynthesis shift from serving as growth and development factors to defence mechanisms against concentration gradient such as those seen in roots of salt stressed plants

## Conclusions

Salinity stress has been demonstrated to be one of the major factors that decreases rice yield produced around the world. However, analysis of the physiological and metabolite changes in rice plant toward salinity stress was fairly limited. In this article, we focused on rice hybrid lines with contrasting behaviours in response to salt stress.

We demonstrated that tolerant line managed to maintain its water content and chlorophyll content even with lower incidence of sodium ion accumulation. In addition, we demonstrated that SOD plays an important oxidative response in plant stress regulatory in tolerant rice line while APX and CAT were important antioxidants in susceptible line.

Result from NMR analysis were in agreement with physiological studies which show distinct but overlapping metabolite alterations of the two lines toward salt stress. Upregulation of non-polar metabolites and production of sucrose, GABA and acetic acid were observed in tolerant rice, suggesting its important role in salinity adaptation. Glutamine and putrescine were noticeably high in susceptible rice. Coordination of different strategies between tolerant and susceptible rice shows that both plants responded differently after exposure to salinity stress. These findings can assist crop development in terms of developing tolerance mechanisms in rice plants.

## Supporting information

S1 Fig Representative ^1^H-NMR spectra of aqueous extracts obtained from *O*. *sativa* SS1-14 control samples. Key for spectra: Key as listed in S1 Table.
(DOCX)

S2 Fig Representative ^1^H-NMR spectra of chloroform extracts obtained *from O*. *sativa* SS1-14 control samples. Key for spectra: Key as listed in S2 Table.
(DOCX)

S1 Table Metabolites identified from leaf sample of aqueous extract. (DOCX)

S2 Table Metabolites identified from leaf sample of chloroform extract. (DOCX)
