# Supplementary material for: Susceptibility and tolerance of rice crop to salt threat: Physiological and metabolic inspections
Source: PLoS One. 2018 Feb 28;13(2):e0192732. doi: 10.1371/journal.pone.0192732 (PMC5831039; doi:10.1371/journal.pone.0192732)
Supplement: S1 Fig — Key for spectra: Key as listed in S1 Table. (DOCX) [file pone.0192732.s001.docx]

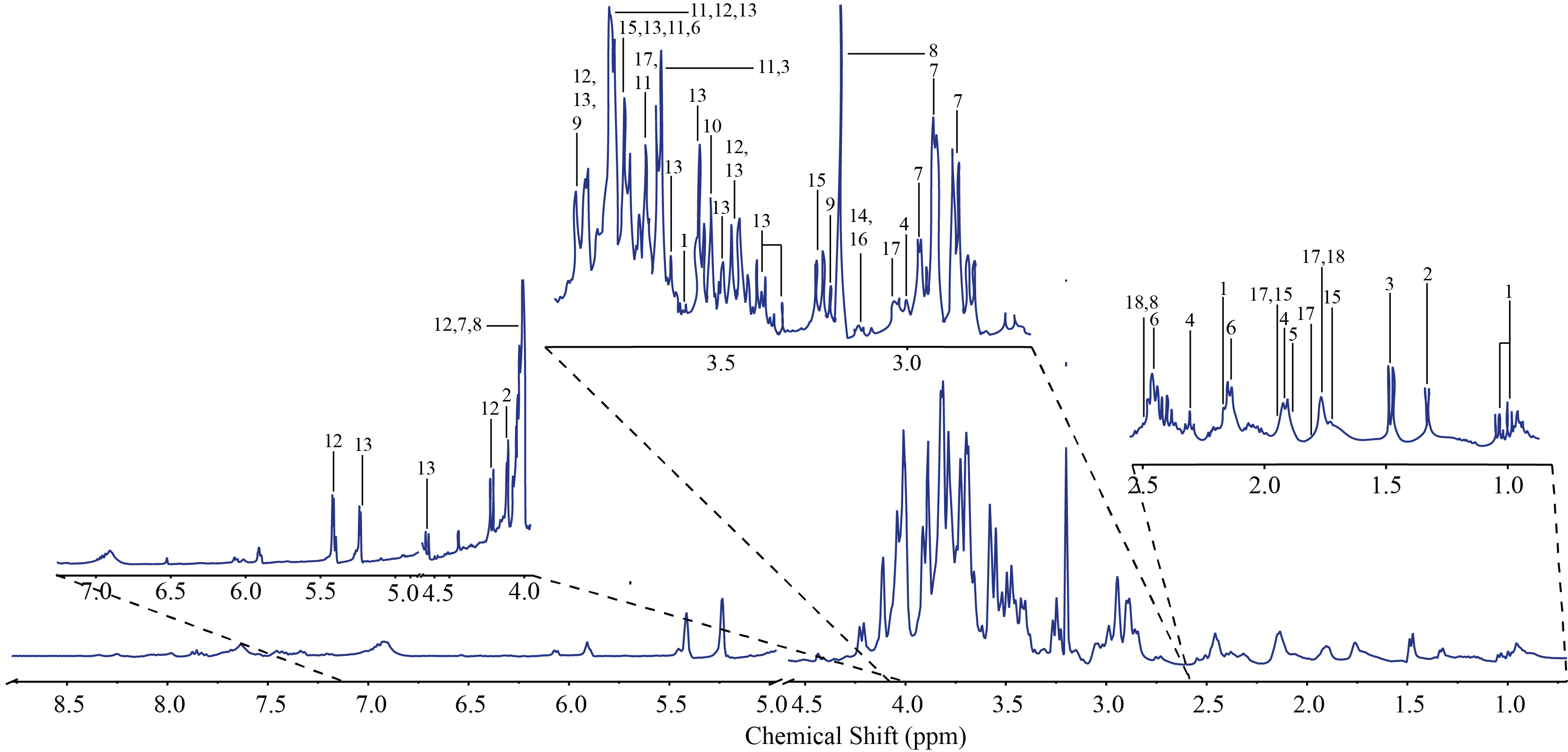


S1 Fig. Representative ^1^H-NMR spectra of aqueous extracts obtained from *O. sativa* SS1-14 control samples. **Key for spectra**: **Key as listed in S1 Table.**
