# Supplementary material for: Susceptibility and tolerance of rice crop to salt threat: Physiological and metabolic inspections
Source: PLoS One. 2018 Feb 28;13(2):e0192732. doi: 10.1371/journal.pone.0192732 (PMC5831039; doi:10.1371/journal.pone.0192732)
Supplement: S1 Table — (DOCX) [file pone.0192732.s003.docx]

**S1 Table. Metabolites identified from leaf sample of aqueous extract.**

| **Metabolites** | **H Group** | **Multiplicity** | **2D COSY cross-peak** |
| --- | --- | --- | --- |
| 1. Valine | CH_3_,  CH_3_,  CHCH_3_,  CHCOOH | 0.98(d),  1.03(d),  2.26(m),  3.60(d) | (1.0,2.25)  (1.0,3.65) |
| 2. Lactic Acid | CH_3_CH,  CHOH | 1.32(d),  4.10(q) | (1.34,4.19) |
| 3. Alanine | CH_3_,  CHCOOH | 1.47(d),  3.68(q) | (1.5,3.72) |
| 4. γ-amino butyric acid | CH_2_,  CH_2_COOH,  CH_2_NH_2_ | 1.89(m),  2.29(t),  3.00(t) | (1.87,2.32)  (1.87,3.01) |
| 5. Acetic acid | CH_3_COOH | 1.91(s) | (1.92,1.92) |
| 6. Glutamine | CH_2_,  CH_2_CONH_2_,  CH(COOH)NH_2_ | 2.13(m),  2.44(m),  3.77(t) | (2.12,2.50)  (2.12,3.75) |
| 7. Asparagine | CH(NH_2_)COOH,  CH_2_CONH_2_, | 4.03(dd),  2.94(m), | (2.92,4.01) |
| 8. Choline | CH_3_,  CH_2_CONH_2_,  CH_2_OH | 3.20(s),  2.53(dd),  4.05(ddd) | (3.20,2.51)  (3.20,4.01) |
| 9. Betaine | CH_2_N^+^(CH_3_)_3_,  CH_3_ | 3.26(s),  3.89(s) | (3.25,3.91) |
| 10. Glycine | CH^2^(COOH)NH^2^ | 3.53(s) | (3.50,3.50) |
| 11. Mannitol | CH_2_OH,  CH_2_OH,  CH_2_OH,  CH_2_OH, | 3.69(dd),  3.75(m),  3.79(dd),  3.83(dd) | (3.70,3.76)  (3.70,3.81)  (3.70,3.85) |
| 12. Sucrose | CHOH,  CHOH,  CHOH,  CHOH,  CHOH,  CHCH_2_OH,  CHCH_2_OH,  CHOH,  CHOH,  CHO | 3.46(t),  3.56(dd),  3.67(s),  3.77(t),  3.82(m),  3.87(dd),  3.89(dd),  4.04(t),  4.20(d),  5.40(d) | (3.52,3.58)  (3.52,3.80)  (3.52,3.91)  (3.52,4.05)  (3.52,4.21)  (3.52,5.42) |
| 13. α-glucose | CHOH,  CHOH,  CHOH,  CHOH,  CHOH,  CHCH_2_OH,  CHOH,  CHOH, | 3.25(dd),  3.40(m),  3.46(m),  3.52(dd),  3.75(m),  3.90(dd),  4.63(d),  5.22(d), | (3.30,3.41)  (3.30,3.51)  (3.30,3.92)  (3.30,4.70)  (3.30,5.25) |
| 14. Malonic acid | CH_2_COOH | 3.11(s) | (3.13,3.13) |
| 15. Argenine | CH_2_,  CH_2_,  CH_2_NH,  CHNH_2_ | 1.68(m),  1.90(m),  3.24(t),  3.75(t) | (1.70,1.93)  (1.70,3.30)  (1.70,3.81) |
| 16. Putrescine | CH_2_,  CH_2_NH_2_ | 1.76(m)(bs),  3.08(t)(bs) | (1.77,3.10) |
| 17. Ornithine | CH_2_,  CH_2_,  CH_2_,  CH_2_NH_2_,  CH(NH_2_)COOH | 1.76(m),  1.83(m),  1.93(m),  3.02(t),  3.73(t) | (1.77,1.85)  (1.77,2.01)  (1.77,3.03)  (1.77,3.81) |
| 18. Trimethylamine | CH_3_N(CH_3_)_2_ | 2.92(s) | (2.85,2.85) |
