# Supplementary material for: Susceptibility and tolerance of rice crop to salt threat: Physiological and metabolic inspections
Source: PLoS One. 2018 Feb 28;13(2):e0192732. doi: 10.1371/journal.pone.0192732 (PMC5831039; doi:10.1371/journal.pone.0192732)
Supplement: S2 Table — (DOCX) [file pone.0192732.s004.docx]

**S2 Table. Metabolites identified from leaf sample of chloroform extract.**

| **Metabolites** | **H Group** | **Multiplicity** | **2D COSY cross-peak** |
| --- | --- | --- | --- |
| 19. Stigmasterol | CH_3_-18, CH(OH)-3,  CH_2_-4,  CH_2_-2,1, CH=CH-5,  CH_2_-22,23,  CH_2_-25 | 0.74 (d),  3.5 (m),  2.21 (m),  (1.8 (m), 1.55 (m), 1.04 (m)),  5.35 (m), | (0.75,1.03)  (0.75,1.56)  (0.75,2.25)  (2.25,3.51) |
| 20. β-sitosterol | CH_3_-18, CH(OH)-3,  CH_2_-4,  CH_2_-2,1, CH=CH-5,  CH_2_-22,23,  CH_2_-25 | 0.69 (d),  3.5 (m),  2.21 (m),  (1.8 (m), 1.55 (m), 1.04 (m)),  5.35 (m), 0.99 (m), 1.64 (m) | (0.69,2.21)  (0.69,1.68)  (1.8,5.35)  (1.0,3.5) |
| 21. Total Fatty Acid | n-CH_3_, (-CH_2_-)n, CH_2_CH_2_COO^-^, CH_2_CH=CH…CH=CHCH_2_, CH_2_COO^_^,  -CH=CH(CH_2_CH=CH)n,  -CH=CH- | 0.87 (m),  1.25 (m),  0.97 (m),  2.05 (m),  2.34 (m),  2.81 (m),  5.43 (m) | (1.25,0.86)  (2.05,1.0)  (1.26,2.35)  (5.40,2.81) |
| 22. PUFA (Polyunsaturated Chain) | =CHCH_2_CH= | 0.955-1.035 (bs) | (0.96,1.03) |
| 23. Alkane Chains | CH_2_, CH_3_ | 0.88(s), 1.25(s) | (0.86,1.25) |
| 24. Lipid Fraction (Choline Head/ phosphatidylcholine) | -OCH_2_CH_2_N^+^ | 3.58 (bs),  4.25 (bs) | (3.58,4.25) |
| 25. Lipid Fraction (triacylglyceride; TAG) | CH sn1,3(a),  CH sn1,3(b),  CH sn2 | 4.31 (m),  4.14 (m),  5.26 (m), | (4.40,5.30) |
| 26. Lipid Fraction  (diglyceride; DAG) | CH sn1,3(a) | 5.13 (m) | (5.14,5.14) |
